# Dating first cases of COVID-19

**DOI:** 10.1371/journal.ppat.1009620

**Published:** 2021-06-24

**Authors:** David L. Roberts, Jeremy S. Rossman, Ivan Jarić

**Affiliations:** 1 Durrell Institute of Conservation and Ecology, School of Anthropology & Conservation, Marlowe Building, University of Kent, Canterbury, Kent, United Kingdom; 2 School of Biosciences, University of Kent, Canterbury, Kent, United Kingdom; 3 Research-Aid Networks, Chicago, Illinois, United States of America; 4 Biology Centre of the Czech Academy of Sciences, Institute of Hydrobiology, České Budějovice, Czech Republic; 5 University of South Bohemia, Faculty of Science, Department of Ecosystem Biology, České Budějovice, Czech Republic; Icahn School of Medicine at Mount Sinai, UNITED STATES

## Abstract

Questions persist as to the origin of the COVID-19 pandemic. Evidence is building that its origin as a zoonotic spillover occurred prior to the officially accepted timing of early December, 2019. Here we provide novel methods to date the origin of COVID-19 cases. We show that six countries had exceptionally early cases, unlikely to represent part of their main case series. The model suggests a likely timing of the first case of COVID-19 in China as November 17 (95% CI October 4). Origination dates are discussed for the first five countries outside China and each continent. Results infer that SARS-CoV-2 emerged in China in early October to mid-November, and by January, had spread globally. This suggests an earlier and more rapid timeline of spread. Our study provides new approaches for estimating dates of the arrival of infectious diseases based on small samples that can be applied to many epidemiological situations.

## Author summary

While the COVID-19 pandemic continues, questions still persist as to its origins. Evidence is building that its origin as a zoonotic spillover occurred before the officially accepted timing of early December, 2019. We date the origin of COVID-19 cases from 203 countries and territories using a model from conservation science. We use a method that was originally developed to date the timing of extinction, and turn it to date the timing of origination using case dates rather than sighting events. Our results suggest that the virus emerged in China in early October to mid-November, 2019 (the most likely date being November 17), and by January, 2020, had spread globally. This suggests a much earlier and more rapid spread than is evident from confirmed cases. In addition, our study provides a new approach for estimating dates of the arrival of infectious diseases in new areas that can be applied to many different situations in the future.

## Introduction

Uncertainty still persists around the origin of severe acute respiratory syndrome coronavirus 2 (SARS-CoV-2) and the resulting COVID-19 disease. While an origin as a zoonotic spillover in the Huanan Seafood Market, Wuhan, sometime during early December, 2019, has been proposed [1], this has been called into question [2–4]. This uncertainty arises due to both the presence of earlier potential COVID-19 cases, and the fact that most phylogenetic analyses put the most recent ancestor at between mid-November and early December, 2019 [5].

Uncertainty around origination dates extends beyond the suggested zoonotic overspill in China to all countries where SARS-CoV-2 has spread. For example, in France the first case of COVID-19 was recorded as January 25, 2020, however a recent retrospective review of medical records from patients in intensive care unit (ICU) with both influenza-like illness (ILI) symptoms and pulmonary ground-glass opacity admitted between December 2, 2019, and January 16, 2020, (14 patients of 58) identified one patient as having COVID-19 who had been presented to the emergency ward on December 27 [6]. In the United States, SARS-CoV-2 RNA was detected through retrospective RT-PCR testing of a woman who had become ill on January 31, 2020, and died on the February 6, 2020, over 3 weeks before the first recognised case on February 26 [7].

Here we repurpose extinction models from conservation science to estimate the potential for earlier cases than has been reported of COVID-19 in 203 countries and territories. Further, we examine exceptionally early cases to determine the likelihood that these cases contributed to the country’s current infection or if they were isolated outbreaks that did not lead to the current lineage of cases. As such we specifically date the origin of cases that resulted in the virus taking hold in each country.

Within the discipline of conservation science, a number of models have been developed to infer or date extinction events based on a series of sightings of a species. Interest lies in determining whether a species still persists, having not been sighted for a period of time. If it is assumed the species is extinct, interest then lies in determining when extinction occurred. The application of these models has been proposed in a number of areas beyond extinction modelling to determine end points, particularly the Optimal Linear Estimation (OLE) method developed by Roberts and Solow [8], including geological stratigraphy [9], archaeology [10], phenological studies [11], and phylogenetics [12]. Based on a series of COVID-19 cases, interest lies in dating the original case. Such a knowledge is critical for our understanding of the spread of this disease.

## Results

**Fig 1:**
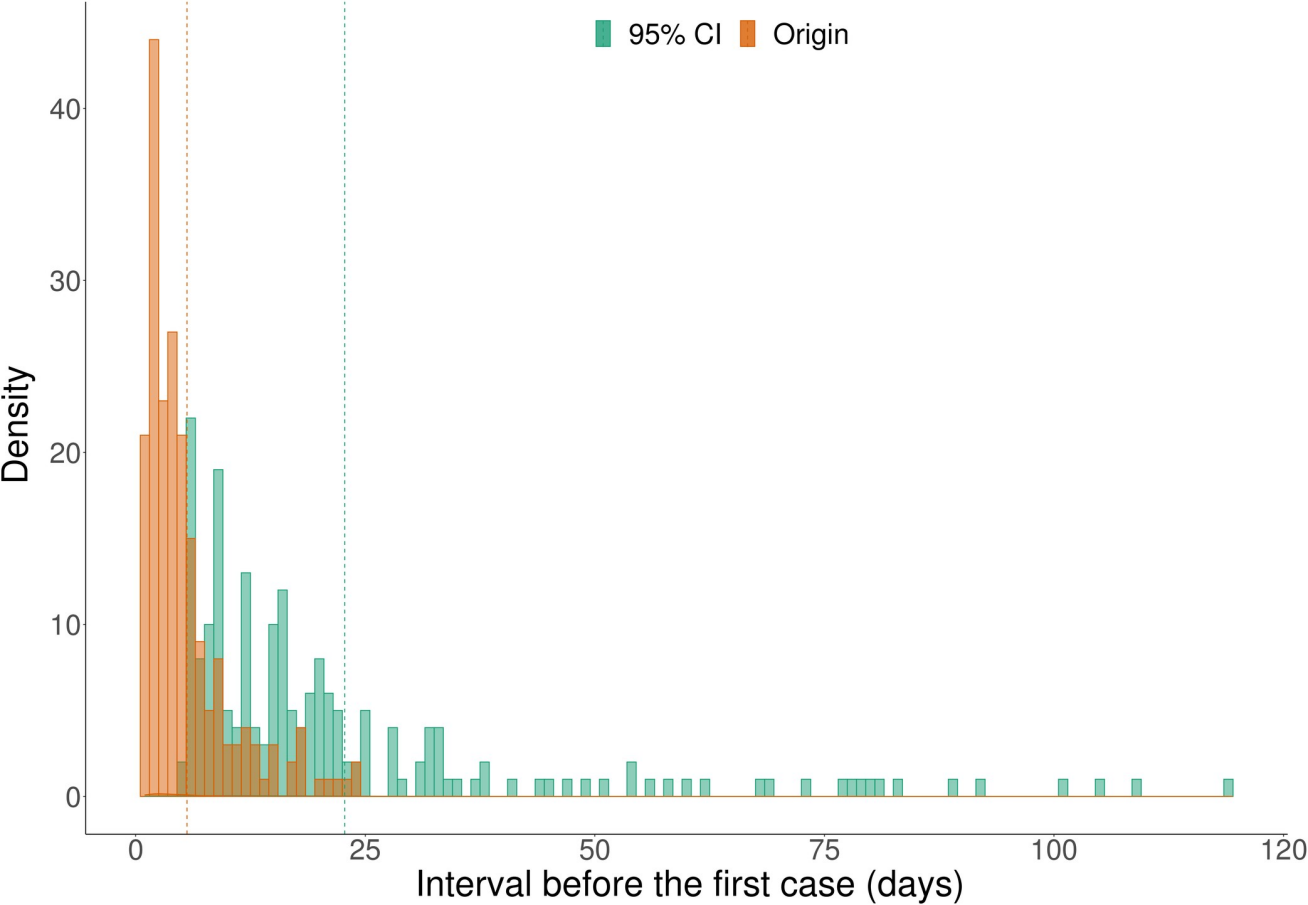
Histogram of intervals between estimated origin dates and first cases (Origin) and between upper bounds of 95% confidence intervals and first cases (95% CI). Vertical dashed lines represent mean values.

As of May 5, 2020, when the Worldometer ‘COVID-19 Coronavirus Pandemic’ [13] dataset was downloaded, there were 203 countries and territories with 5 or more case dates. When the surprise method [14] was applied, six countries (Belgium, Cambodia, Italy, Russia, Sri Lanka, Sweden) had cases that were exceptionally early and unlikely to represent part of the main dataset (p<0.05; S1 Table). A further three countries were significant at p<0.10 (Finland, Nepal, Yemen). Such case dates were removed from the dataset, while maintaining *k* at between 5 and 10 depending on the number of available case dates. By removing the earliest case date from the record for Yemen the number of case dates fell below five and therefore Yemen was not analysed. Using the OLE, origination dates were 4.2 days before the first verified case (median value), with Timor and Sint Maarten having the longest extension on the origination date of 24 days (Fig 1).

**Fig 2:**
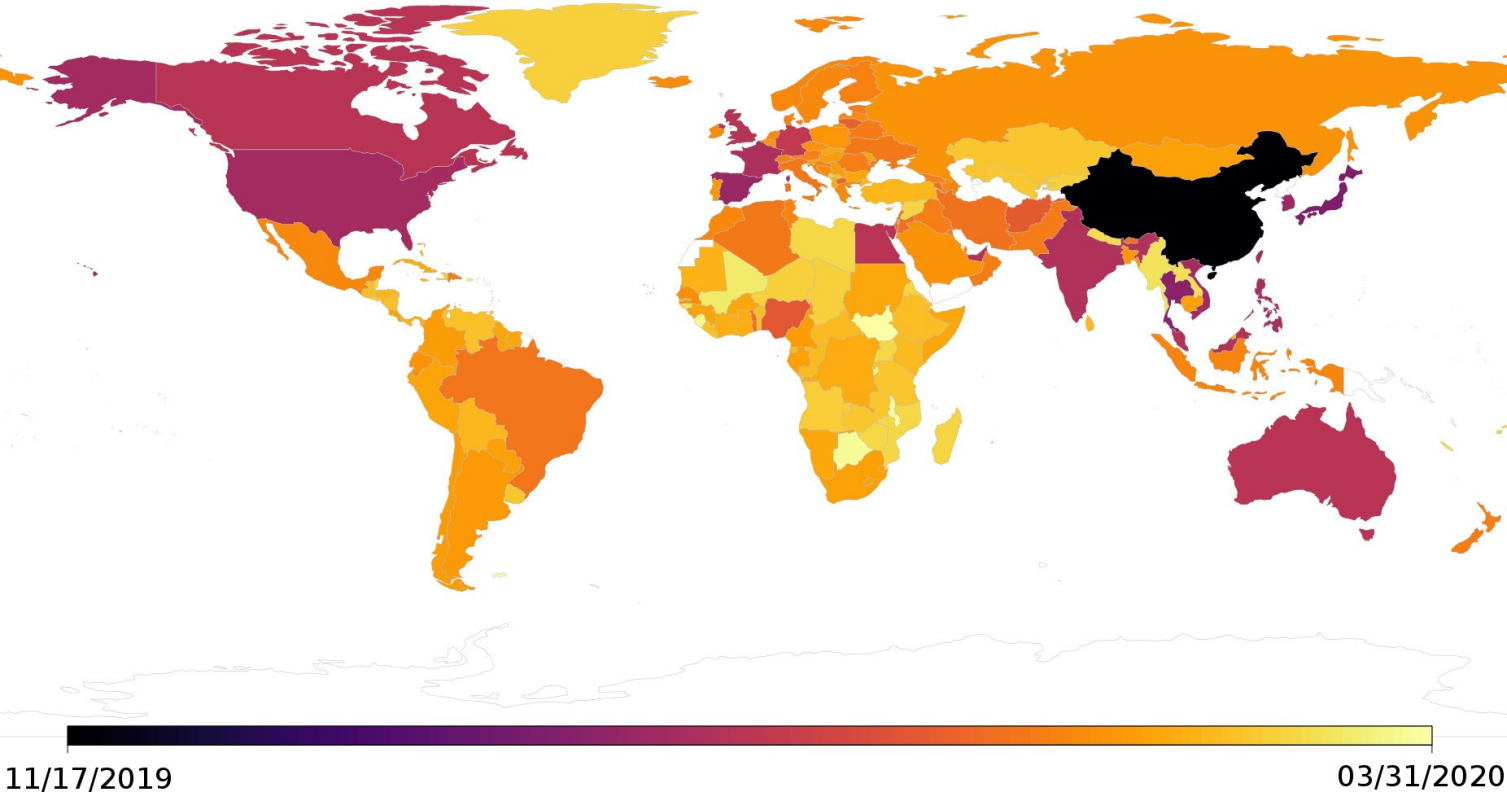
Map of the estimated origin dates per country. Map layers were created using the R package rworldmap, Version 1.3–6 (http://cran.r-project.org/web/packages/rworldmap).

Based on the OLE, the results suggest that the most likely timing of the first case of COVID-19 in China was November 17, 2019 (Fig 2), with a 95% confidence interval reaching as far back as October 4.

The results suggest that the virus spread beyond China by January 2020 with the estimated first case being in Japan on January 3, 2020 (95% CI November 29, 2019), and followed by Thailand on January 7, 2020 (95% CI December 22, 2019). The third earliest origination date, outside of China, suggests that the virus had left eastern Asia and arrived in Europe, with an estimated first case on January 12, 2020 (95% CI November 3, 2019), in Spain (however see result for Italy presented below). The virus appears to have continued spreading to other countries in eastern Asia with the fourth earliest origination date outside of China being in South Korea on January 14, 2020 (95% CI December 31, 2019). Following the spread to Europe (third earliest origination date), the virus appears to have spread to North America with an estimated first case being in the United States on January 16, 2020 (95% CI January 3, 2020), making the United States the fifth country.

Beyond the earliest five cases outside of China, as mentioned previously, the estimated first introduction to another continent was in Europe on January 12, 2020 (95% CI November 3, 2019) in Spain (however see result for Italy presented below), and in North America on January 16, 2020 (95% CI January 3, 2020) in the United States. Estimated first cases on other continents are in Australia on January 23, 2020 (95% CI January 16, 2019), Africa on February 9, 2020 (95% CI December 8, 2019) in Nigeria, and South America on February 19, 2020 (95% CI February 2, 2020) in Brazil (Fig 2 and S2 Table and S1 Video).

Additional notable results are the estimated dates within Europe. As mentioned previously, the estimated first introduction to Europe was on January 12, 2020 (95% CI November 3, 2019) in Spain and on January 19, 2020 (95% CI January 4, 2020) in France. The results suggest this was followed by the United Kingdom on January 22, 2020 (95% CI December 30, 2019), Germany on January 26, 2020 (95% CI January 21, 2020), followed by Monaco, Lithuania, Vatican, and Macedonia, between February 12–14, 2020, and Italy not until February 20, 2020 (95% CI February 16, 2020). However, it should be noted that Italy was one of the six countries with exceptionally early cases and therefore the result for Italy was affected by the removal of this early case (i.e. on January 31, 2020; S1 Table). If this significantly early case is included then the estimated first case is January 1, 2020 (95% CI August 10, 2019; S3 Table).

## Discussion

While the first case of COVID-19 was officially identified in early December, 2019 [1], it is likely that SARS-CoV-2 had spilled over into humans much earlier. Nsoesie et al. [3] identified significant changes in hospital and search engine traffic in Wuhan during August to October, 2019, suggesting a possible earlier existence of COVID-19. The recent joint WHO-China study on the global origin of SARS-CoV-2 found that, based on a review of molecular evidence, most point estimates place the most recent ancestor at between mid-November and early December, with a range from late September to early December [5]. Our results support the existing evidence and suggest that the first case of COVID-19 would have been sometime between early October and mid-November. Further, our results suggest the most likely timing of the first case to be November 17, 2019.

Expanding this comparison to four other studies that identified earlier cases, we inferred January 7, 2020 (95% CI December 22, 2019) as the most likely first case in Thailand. This is only 1 day after a case identified in a traveller to Thailand from Wuhan on January 8, 2020 [15,16]. However, the most likely first case in France was inferred as January 19, 2020 (95% CI January 4, 2020), while a retrospective review of medical records identified one patient as having COVID-19 from December 27 [6]. This is eight days earlier than our inferred 95% CI. Similarly, from an analysis of 40 composite influent wastewater samples from northern Italy, La Rosa et al. [17] detected SARS-CoV-2 in samples collected on December 18, 2019, over six weeks before the first confirmed case on January 31, 2020. In our analysis we removed this first case (January 31, 2020) as it was significantly divergent in terms of timing from the rest of the cases, resulting in an inferred first case of February 20, 2020 (95% CI February 16, 2020). However, if this first case is retained then it pushes back our inferred first case to January 1, 2020 (95% CI August 10, 2019), which is more in line with the findings of La Rosa et al. [17]. In contrast, our analysis pushed back the most likely first case in the United States to mid-January (January 16, 2020; 95% CI January 3, 2020), two weeks prior to the earliest known case of a woman identified through retrospective testing who became ill on January 31, 2020, and almost 6 weeks before the first recognised case on February 26 [7]. Further analysis of retrospective testing studies will help validate the application of OLE and associated methods.

Using the method of Solow and Smith [14], we identified six countries with exceptionally early cases of COVID-19 compared with the rest of the case time series for those countries. These may represent isolated cases, infections that did not contribute to the eventual spread of COVID-19 through the country or territory. However, currently only the results of retrospective testing have been published for Italy as described above. Without such analyses it is not possible to determine if our results have in fact identified early isolated cases or simply reflect poor surveillance and pre-symptomatic transmission.

In the same way the extinction events are rarely observed, so too are origination events such as those of COVID-19. Without rigorous tracing systems, dating the first cases has to be inferred. In the case of emerging infectious diseases, this is most frequently based on phylogenetic analysis. For this to be meaningful, it requires sufficient sampling and diversity. Here we applied a well-established extinction estimator (i.e. OLE) from conservation science, to estimate the origination times of COVID-19 for all countries with five or more case dates. As the method can be effectively applied to very sparse datasets, with as few as 4–5 records [18,19], it illustrates the potential to rapidly gain an understanding of the origination timings of novel zoonotic diseases when they are poorly known. Moreover, some of the approaches from this group of methods can be applied even to records with just two [20] or even a single record [21]. Using methods borrowed from conservation science, we are able to estimate a range of likely dates for the zoonotic spillover of COVID-19 into humans in China and the subsequent spread to countries around the world.

## Materials and methods

Here we estimated the timing of the origination of COVID-19 in multiple countries using the publicly-available Worldometer ‘COVID-19 Coronavirus Pandemic’ dataset [13]. As the dataset of cases in China does not extend to the first verified cases, we used the dataset presented by Huang et al. [1]. From these datasets we created time series of new cases for each country. While the datasets present the number of cases per day, it is not possible to determine whether these cases are independent or related. We therefore used the case days rather than individual cases (i.e. multiple cases on the same day are treated as a single case day); this is standard practice within conservation science when considering sightings of potentially extinct species [e.g. 18,22]. It is important to note that case days represent the time when cases were reported, and not the time of transmission.

### Testing for exceptionally early cases that failed to take hold

There are a number of exceptionally early cases in specific countries that may have arisen for a number of reasons (e.g. repatriation) and may represent isolated cases that did not contribute to the eventual spread throughout the country [e.g. 23]. These exceptionally early cases propagate uncertainty in origination estimators and therefore we applied a method proposed by Solow and Smith [14] to identify such cases. In the context of COVID-19, this method asks the question, given an early case, what is the probability it belongs to the main body of cases? This method has been previously used in conservation science to determine whether new sightings of the European polecat (*Mustela putorius*) in Scotland arose from the native population that was thought to be extirpated or arose from surreptitious reintroduction [20]. Here we use this method to identify cases of COVID-19 that appear not to have taken hold within a country.

Let *t*_1_>*t*_2_>…>*t*_*k*_ be the *k* earliest cases of COVID-19, ordered from the most recent to the earliest case. The basic assumption of Solow and Smith [14] method is that these represent the *k* largest values of a larger collection of values generated from a distribution from the Gumbel domain of attraction. Suppose that an earlier case of COVID-19 is recorded at time *y*, interest centres on assessing the exceptionality of the earlier record. Solow and Smith [14] showed that, under the null hypothesis that the new record was generated by the same process as the earlier ones, the quantity,

$$S_{k}=\frac{y-t_{1}}{(y-t_{1})+\sum_{j=1}^{k-1}\left(j+1)(t_{j}-t_{j+1}\right)},$$

has a *β* distribution with parameters 1 and *k*-1 so that the p-value corresponding to an observed value *S*_*k*_ is

$$p={\left(1‐s_{k}\right)}^{k‐1}.$$

We applied this test using the first 5 to 10 (*k*) earliest case dates of COVID-19 depending on the length of the case record for each country.

### Origination estimator

We applied the Optimal Linear Estimation (OLE) method as proposed by Roberts and Solow [8] for dating extinctions. OLE uses the time series of last known chronological occurrences of the studied phenomenon to estimate the time after the last known occurrence when the process that was generating them has stopped, and the phenomenon will consequently no longer be observable. However, in our case we are interested in the timing of origination rather than extinction, so we apply it here with the reverse temporal direction [10]. The OLE method has proved to be robust in the inference of extinction under a variety of scenarios, reporting probabilities and trends [18,24]. It is important to note that underlying assumptions of the OLE are not specific to biological organisms and the species extinction process, and that the method does not contain any biologically specific parameters. OLE simply takes into account intervals between occurrences of a phenomenon and their distribution, irrespective of the type of phenomenon studied. This makes it readily applicable to diverse types of phenomena, as long as they are characterized by sporadic records made before the phenomenon or the process ceased [10]. It also does not require a complete record, but it accounts for records being generated based on some unknown probability. OLE has been shown to perform well under different rates and trends in sighting effort [18,24], which in this case corresponds to reporting probability. Furthermore, OLE is a non-parametric method and it does not make any assumptions about the sighting rates or data distribution, making it more flexible compared to other methods [19,25]. Finally, OLE is based on extreme value theory, which shows that the distribution of the maximum is well approximated by the generalised extreme value distribution, regardless of the actual distribution of records [19,25,26].

Let *T*_1_>*T*_2_>…>*T*_k_ be the *k* earliest case dates of COVID-19, ordered from the most recent (with *T*_*k*_ being the first known case date). Interest centres on using this record of case dates to estimate the origination time, *θ*. In this context, optimal linear estimation is based on the fact that the joint distribution of the *k* earliest case dates has the same approximate ‘Weibull form’, regardless of the parent distribution of the complete record of case dates [8,19].

The optimal linear estimator of *θ* has the form of a weighted sum of the case date times,

$$\hat{\theta }=\sum_{i-1}^{k}a_{i}T_{i}$$

The vector of weights is given by

$$a={\left(e^{t}\wedge^{-1}e\right)}^{-1}\wedge^{-1}e$$

where *e* is a vector of *k* 1’s and ∧ is the symmetric *k*×*k* matrix with typical element $\lambda_{ij}=\frac{\Gamma (2\hat{v}+i)\Gamma (\hat{v}+j)}{\Gamma (\hat{v}+i)\Gamma (j)}$, *j*≤*i*, and where *Γ* is the standard gamma function. Also,

$$\hat{v}=\frac{1}{k-1}\sum_{i-1}^{k-2}log\frac{T_{1}-T_{k}}{T_{1}-T_{i+1}}$$

is an estimate of the shape parameter of the joint Weibull distribution of the *k* earliest case date times. Following Solow [19], an approximate one-sided upper bound of a 1−*α* confidence interval (CI) for *θ* is

$$S_{U}=\frac{t_{n}-c(\alpha )t_{n-k+1}}{1-c(\alpha )},$$

where $c(\alpha )={\left(\frac{k}{-log\alpha }\right)}^{-\hat{v}}$; note that in Solow [19] the equation for *c*(*α*) was incorrectly inverted.

Having excluded exceptionally early cases using the method of Solow and Smith [14], as they likely represent cases where COVID-19 has failed to take hold, we used the first 5 to 10 (*k*) earliest confirmed case dates for each country as suggested by Solow [19] and Rivadeneira et al. [24]. As such we date the origin of COVID-19 that gave rise to the main spread within a country. However, as there is no specific start date as it varies depending on the arrival time of COVID-19 in each country, the 10th case date is used as the end of the period. The origination date was calculated using the R software package sExtinct [27].

OLE has been widely used in diverse scientific fields, and it is recognized as the most robust approach within that family of methods [18,24,28]. It has demonstrated high levels of accuracy of its predictions in the majority of scenarios, especially in case of declining and low record frequency, while its flexibility and non-parametric nature allow its wide application for various data types and conditions [18,24,25,28,29].

It is important, however, to acknowledge some limitations of the presented approach, related to the input data quality and reliability. While OLE was demonstrated to be robust to limited data [18,24], as with any methods, here the predictions of the method are only as good as the data used, and the diligence and quality of COVID-19 testing and reporting within different countries is likely to affect our results. There are considerable differences among countries and regions in the COVID-19 testing rates, surveillance effort, indicators, reporting systems and criteria, and data quality [30]. The method also does not account for individual differences among characteristics among records in quality or reliability [31]. While one solution for this issue would be to apply weighted resampling method [32], which allows OLE to effectively incorporate individual reliability of records, detailed analysis of official covid records necessary to develop such reliability scoring is beyond the scope of this study. Furthermore, the differences among countries in implemented control measures, such as travel bans, could also affect inferred spatiotemporal dynamics of COVID-19 spread. In addition, inferences of the origination date may be potentially affected by multiple COVID-19 introduction events within a country. The applied ‘surprise method’ by Solow and Smith [14] identified exceptionally early, isolated records that may not represent part of the main dataset, being introductions of the virus that did not lead to sustained transmission chains. However, potential multiple introduction events temporally situated within the main cluster of records will not be distinguished by such methods. Finally, dates of records used in the analysis represent mostly the time when a case was reported, not the actual time of transmission and infection, and the results of the analysis consequently also represent a hypothetical date when the symptoms of the first, unreported case became manifested. Consequently, if interest lies in inferring the time of first transmission, origin date should be pushed further in the past for the duration of the incubation period. Bearing in mind all of the limitations of the COVID-19 records data and the method used in the study, these potential issues need to be taken into consideration when interpreting the results.

## Supporting information

S1 Table Results of the testing of exceptionally early cases per country using the method of Solow & Smith.^1^. (DOC)

S2 Table Results of the COVID-19 origin dating per country, based on the Optimal Linear Estimation method.^1^ *N*—number of case dates used for the analysis, *t_k_*—duration of the interval between the first and the last case date used in the analysis (days), *θ* - estimated origin date, expressed as the number of days before the earliest reported case date, *S_U_*—upper bound of a 95% confidence interval of the estimated origin date. (DOC)

S3 Table Results of the COVID-19 origin dating without the correction of the first case date, for the nine countries that had their first case dates adjusted (see Methods for information). *N*—number of case dates used for the analysis, *t*_*k*_—duration of the interval between the first and the last case date used in the analysis (days), *θ* - estimated origin date, expressed as the number of days before the earliest reported case date, *S*_*U*_—upper bound of a 95% confidence interval of the estimated origin date.
(DOC)

S1 Video Video presents global COVID-19 spread across countries over time. Countries marked in yellow—upper bound of a 95% confidence interval of the estimated origin date includes particular date (i.e., probability of the country already experiencing first case is above 5%); countries marked in orange—estimated origin date already occurred by that date (i.e. origin dating indicates that the COVID-19 is already spreading in the country); countries marked in red—first reported case already occurred by that date; countries marked in grey—insufficient data for origin dating. Map layers were created using the R package rworldmap, Version 1.3–6 (http://cran.r-project.org/web/packages/rworldmap).
(MOV)
