# Supplementary material for: Dating first cases of COVID-19
Source: PLoS Pathog. 2021 Jun 24;17(6):e1009620. doi: 10.1371/journal.ppat.1009620 (PMC8224943; doi:10.1371/journal.ppat.1009620)
Supplement: S1 Table — (DOC) [file ppat.1009620.s001.doc]

**S1 Table.** Results of the testing of exceptionally early cases per country using the method of Solow & Smith.^1^

| **No** | **Entity** | **Code** | **First case date tested** | ***P* of first sighting** | **First case excluded** | **Corrected first case date** |
| --- | --- | --- | --- | --- | --- | --- |
| 1 | Afghanistan | AFG | Feb 25, 2020 | 0.323 | No | Feb 25, 2020 |
| 2 | Albania | ALB | Mar 9, 2020 | 0.835 | No | Mar 9, 2020 |
| 3 | Algeria | DZA | Feb 26, 2020 | 0.663 | No | Feb 26, 2020 |
| 4 | Andorra | AND | Mar 3, 2020 | 0.238 | No | Mar 3, 2020 |
| 5 | Angola | AGO | Mar 22, 2020 | 0.553 | No | Mar 22, 2020 |
| 6 | Antigua and Barbuda | ATG | Mar 15, 2020 | 0.721 | No | Mar 15, 2020 |
| 7 | Argentina | ARG | Mar 4, 2020 | 0.751 | No | Mar 4, 2020 |
| 8 | Armenia | ARM | Mar 1, 2020 | 0.168 | No | Mar 1, 2020 |
| 9 | Aruba | ABW | Mar 13, 2020 | 0.499 | No | Mar 13, 2020 |
| 10 | Australia | AUS | Jan 25, 2020 | 0.934 | No | Jan 25, 2020 |
| 11 | Austria | AUT | Feb 26, 2020 | 0.701 | No | Feb 26, 2020 |
| 12 | Azerbaijan | AZE | Feb 29, 2020 | 0.835 | No | Feb 29, 2020 |
| 13 | Bahamas | BHS | Mar 16, 2020 | 0.633 | No | Mar 16, 2020 |
| 14 | Bahrain | BHR | Feb 24, 2020 | 0.883 | No | Feb 24, 2020 |
| 15 | Bangladesh | BGD | Mar 9, 2020 | 0.350 | No | Mar 9, 2020 |
| 16 | Barbados | BRB | Mar 18, 2020 | 0.776 | No | Mar 18, 2020 |
| 17 | Belarus | BLR | Feb 28, 2020 | 0.612 | No | Feb 28, 2020 |
| 18 | Belgium | BEL | Feb 4, 2020 | 0.022 | Yes | Mar 2, 2020 |
| 19 | Belize | BLZ | Mar 24, 2020 | 0.801 | No | Mar 24, 2020 |
| 20 | Benin | BEN | Mar 17, 2020 | 0.795 | No | Mar 17, 2020 |
| 21 | Bermuda | BMU | Mar 20, 2020 | 0.742 | No | Mar 20, 2020 |
| 22 | Bhutan | BTN | Mar 6, 2020 | 0.605 | No | Mar 6, 2020 |
| 23 | Bolivia | BOL | Mar 12, 2020 | 0.866 | No | Mar 12, 2020 |
| 24 | Bonaire Sint Eustatius and Saba | BES | Apr 2, 2020 | 0.370 | No | Apr 2, 2020 |
| 25 | Bosnia and Herzegovina | BIH | Mar 6, 2020 | 0.467 | No | Mar 6, 2020 |
| 26 | Botswana | BWA | Apr 1, 2020 | 0.952 | No | Apr 1, 2020 |
| 27 | Brazil | BRA | Feb 26, 2020 | 0.602 | No | Feb 26, 2020 |
| 28 | British Virgin Islands | VGB | Mar 27, 2020 | 0.866 | No | Mar 27, 2020 |
| 29 | Brunei | BRN | Mar 10, 2020 | 0.701 | No | Mar 10, 2020 |
| 30 | Bulgaria | BGR | Mar 8, 2020 | 0.848 | No | Mar 8, 2020 |
| 31 | Burkina Faso | BFA | Mar 11, 2020 | 0.576 | No | Mar 11, 2020 |
| 32 | Burundi | BDI | Apr 1, 2020 | 0.906 | No | Apr 1, 2020 |
| 33 | Cambodia | KHM | Jan 28, 2020 | 0.033 | Yes | Mar 08, 2020 |
| 34 | Cameroon | CMR | Mar 7, 2020 | 0.832 | No | Mar 7, 2020 |
| 35 | Canada | CAN | Jan 26, 2020 | 0.914 | No | Jan 26, 2020 |
| 36 | Cape Verde | CPV | Mar 21, 2020 | 0.938 | No | Mar 21, 2020 |
| 37 | Cayman Islands | CYM | Mar 20, 2020 | 0.880 | No | Mar 20, 2020 |
| 38 | Central African Republic | CAF | Mar 16, 2020 | 0.744 | No | Mar 16, 2020 |
| 39 | Chad | TCD | Mar 20, 2020 | 0.871 | No | Mar 20, 2020 |
| 40 | Chile | CHL | Mar 4, 2020 | 0.848 | No | Mar 4, 2020 |
| 41 | China | CHN | Dec 31, 2019 | 0.297 | No | Dec 31, 2019 |
| 42 | Colombia | COL | Mar 7, 2020 | 0.622 | No | Mar 7, 2020 |
| 43 | Congo | COG | Mar 16, 2020 | 0.843 | No | Mar 16, 2020 |
| 44 | Costa Rica | CRI | Mar 7, 2020 | 0.701 | No | Mar 7, 2020 |
| 45 | Cote d'Ivoire | CIV | Mar 12, 2020 | 0.663 | No | Mar 12, 2020 |
| 46 | Croatia | HRV | Feb 26, 2020 | 0.866 | No | Feb 26, 2020 |
| 47 | Cuba | CUB | Mar 12, 2020 | 0.731 | No | Mar 12, 2020 |
| 48 | Curacao | CUW | Mar 13, 2020 | 0.798 | No | Mar 13, 2020 |
| 49 | Cyprus | CYP | Mar 10, 2020 | 0.766 | No | Mar 10, 2020 |
| 50 | Czech Republic | CZE | Mar 2, 2020 | 0.842 | No | Mar 2, 2020 |
| 51 | Democratic Republic of Congo | COD | Mar 11, 2020 | 0.748 | No | Mar 11, 2020 |
| 52 | Denmark | DNK | Feb 27, 2020 | 0.701 | No | Feb 27, 2020 |
| 53 | Djibouti | DJI | Mar 19, 2020 | 0.460 | No | Mar 19, 2020 |
| 54 | Dominica | DMA | Mar 23, 2020 | 0.859 | No | Mar 23, 2020 |
| 55 | Dominican Republic | DOM | Mar 2, 2020 | 0.608 | No | Mar 2, 2020 |
| 56 | Ecuador | ECU | Mar 1, 2020 | 0.897 | No | Mar 1, 2020 |
| 57 | Egypt | EGY | Feb 15, 2020 | 0.125 | No | Feb 15, 2020 |
| 58 | El Salvador | SLV | Mar 19, 2020 | 0.788 | No | Mar 19, 2020 |
| 59 | Equatorial Guinea | GNQ | Mar 15, 2020 | 0.653 | No | Mar 15, 2020 |
| 60 | Eritrea | ERI | Mar 22, 2020 | 0.670 | No | Mar 22, 2020 |
| 61 | Estonia | EST | Feb 28, 2020 | 0.516 | No | Feb 28, 2020 |
| 62 | Ethiopia | ETH | Mar 14, 2020 | 0.843 | No | Mar 14, 2020 |
| 63 | Faeroe Islands | FRO | Mar 20, 2020 | 0.835 | No | Mar 20, 2020 |
| 64 | Falkland Islands | FLK | Apr 4, 2020 | 0.898 | No | Apr 4, 2020 |
| 65 | Fiji | FJI | Mar 20, 2020 | 0.874 | No | Mar 20, 2020 |
| 66 | Finland | FIN | Jan 30, 2020 | 0.077 | Yes | Feb 27, 2020 |
| 67 | France | FRA | Jan 25, 2020 | 0.821 | No | Jan 25, 2020 |
| 68 | French Polynesia | PYF | Mar 19, 2020 | 0.759 | No | Mar 19, 2020 |
| 69 | Gabon | GAB | Mar 13, 2020 | 0.633 | No | Mar 13, 2020 |
| 70 | Gambia | GMB | Mar 18, 2020 | 0.805 | No | Mar 18, 2020 |
| 71 | Georgia | GEO | Feb 27, 2020 | 0.769 | No | Feb 27, 2020 |
| 72 | Germany | DEU | Jan 28, 2020 | 0.913 | No | Jan 28, 2020 |
| 73 | Ghana | GHA | Mar 13, 2020 | 0.513 | No | Mar 13, 2020 |
| 74 | Gibraltar | GIB | Mar 20, 2020 | 0.693 | No | Mar 20, 2020 |
| 75 | Greece | GRC | Feb 27, 2020 | 0.868 | No | Feb 27, 2020 |
| 76 | Greenland | GRL | Mar 20, 2020 | 0.739 | No | Mar 20, 2020 |
| 77 | Grenada | GRD | Mar 23, 2020 | 0.861 | No | Mar 23, 2020 |
| 78 | Guam | GUM | Mar 19, 2020 | 0.835 | No | Mar 19, 2020 |
| 79 | Guatemala | GTM | Mar 15, 2020 | 0.711 | No | Mar 15, 2020 |
| 80 | Guernsey | GGY | Mar 20, 2020 | 0.644 | No | Mar 20, 2020 |
| 81 | Guinea | GIN | Mar 14, 2020 | 0.494 | No | Mar 14, 2020 |
| 82 | Guinea-Bissau | GNB | Mar 27, 2020 | 0.616 | No | Mar 27, 2020 |
| 83 | Guyana | GUY | Mar 13, 2020 | 0.767 | No | Mar 13, 2020 |
| 84 | Haiti | HTI | Mar 20, 2020 | 0.680 | No | Mar 20, 2020 |
| 85 | Honduras | HND | Mar 12, 2020 | 0.744 | No | Mar 12, 2020 |
| 86 | Hungary | HUN | Mar 5, 2020 | 0.701 | No | Mar 5, 2020 |
| 87 | Iceland | ISL | Feb 29, 2020 | 0.701 | No | Feb 29, 2020 |
| 88 | India | IND | Jan 30, 2020 | 0.840 | No | Jan 30, 2020 |
| 89 | Indonesia | IDN | Mar 2, 2020 | 0.492 | No | Mar 2, 2020 |
| 90 | Iran | IRN | Feb 20, 2020 | 0.835 | No | Feb 20, 2020 |
| 91 | Iraq | IRQ | Feb 25, 2020 | 0.842 | No | Feb 25, 2020 |
| 92 | Ireland | IRL | Mar 1, 2020 | 0.633 | No | Mar 1, 2020 |
| 93 | Isle of Man | IMN | Mar 21, 2020 | 0.872 | No | Mar 21, 2020 |
| 94 | Israel | ISR | Feb 22, 2020 | 0.782 | No | Feb 22, 2020 |
| 95 | Italy | ITA | Jan 31, 2020 | 0.039 | Yes | Feb 22, 2020 |
| 96 | Jamaica | JAM | Mar 12, 2020 | 0.899 | No | Mar 12, 2020 |
| 97 | Japan | JPN | Jan 15, 2020 | 0.260 | No | Jan 15, 2020 |
| 98 | Jersey | JEY | Mar 20, 2020 | 0.905 | No | Mar 20, 2020 |
| 99 | Jordan | JOR | Mar 3, 2020 | 0.126 | No | Mar 3, 2020 |
| 100 | Kazakhstan | KAZ | Mar 15, 2020 | 0.835 | No | Mar 15, 2020 |
| 101 | Kenya | KEN | Mar 14, 2020 | 0.806 | No | Mar 14, 2020 |
| 102 | Kosovo | RKS | Mar 16, 2020 | 0.731 | No | Mar 16, 2020 |
| 103 | Kuwait | KWT | Feb 24, 2020 | 0.899 | No | Feb 24, 2020 |
| 104 | Kyrgyzstan | KGZ | Mar 19, 2020 | 0.894 | No | Mar 19, 2020 |
| 105 | Laos | LAO | Mar 25, 2020 | 0.819 | No | Mar 25, 2020 |
| 106 | Latvia | LVA | Mar 3, 2020 | 0.423 | No | Mar 3, 2020 |
| 107 | Lebanon | LBN | Feb 22, 2020 | 0.562 | No | Feb 22, 2020 |
| 108 | Liberia | LBR | Mar 17, 2020 | 0.924 | No | Mar 17, 2020 |
| 109 | Libya | LBY | Mar 25, 2020 | 0.616 | No | Mar 25, 2020 |
| 110 | Liechtenstein | LIE | Mar 5, 2020 | 0.471 | No | Mar 5, 2020 |
| 111 | Lithuania | LTU | Feb 28, 2020 | 0.168 | No | Feb 28, 2020 |
| 112 | Luxembourg | LUX | Mar 1, 2020 | 0.461 | No | Mar 1, 2020 |
| 113 | Macedonia | MKD | Feb 27, 2020 | 0.344 | No | Feb 27, 2020 |
| 114 | Madagascar | MDG | Mar 21, 2020 | 0.610 | No | Mar 21, 2020 |
| 115 | Malawi | MWI | Apr 3, 2020 | 0.860 | No | Apr 3, 2020 |
| 116 | Malaysia | MYS | Jan 25, 2020 | 0.888 | No | Jan 25, 2020 |
| 117 | Maldives | MDV | Mar 8, 2020 | 0.941 | No | Mar 8, 2020 |
| 118 | Mali | MLI | Mar 26, 2020 | 0.863 | No | Mar 26, 2020 |
| 119 | Malta | MLT | Mar 8, 2020 | 0.711 | No | Mar 8, 2020 |
| 120 | Mauritania | MRT | Mar 15, 2020 | 0.884 | No | Mar 15, 2020 |
| 121 | Mauritius | MUS | Mar 20, 2020 | 0.835 | No | Mar 20, 2020 |
| 122 | Mexico | MEX | Feb 29, 2020 | 0.893 | No | Feb 29, 2020 |
| 123 | Moldova | MDA | Mar 8, 2020 | 0.590 | No | Mar 8, 2020 |
| 124 | Monaco | MCO | Feb 29, 2020 | 0.168 | No | Feb 29, 2020 |
| 125 | Mongolia | MNG | Mar 10, 2020 | 0.631 | No | Mar 10, 2020 |
| 126 | Montenegro | MNE | Mar 18, 2020 | 0.848 | No | Mar 18, 2020 |
| 127 | Montserrat | MSR | Mar 21, 2020 | 0.692 | No | Mar 21, 2020 |
| 128 | Morocco | MAR | Mar 3, 2020 | 0.649 | No | Mar 3, 2020 |
| 129 | Mozambique | MOZ | Mar 23, 2020 | 0.793 | No | Mar 23, 2020 |
| 130 | Myanmar | MMR | Mar 24, 2020 | 0.893 | No | Mar 24, 2020 |
| 131 | Namibia | NAM | Mar 15, 2020 | 0.593 | No | Mar 15, 2020 |
| 132 | Nepal | NPL | Jan 25, 2020 | 0.052 | Yes | Mar 24, 2020 |
| 133 | Netherlands | NLD | Feb 28, 2020 | 0.835 | No | Feb 28, 2020 |
| 134 | New Caledonia | NCL | Mar 21, 2020 | 0.917 | No | Mar 21, 2020 |
| 135 | New Zealand | NZL | Feb 28, 2020 | 0.643 | No | Feb 28, 2020 |
| 136 | Nicaragua | NIC | Mar 19, 2020 | 0.914 | No | Mar 19, 2020 |
| 137 | Niger | NER | Mar 21, 2020 | 0.715 | No | Mar 21, 2020 |
| 138 | Nigeria | NGA | Feb 28, 2020 | 0.271 | No | Feb 28, 2020 |
| 139 | Northern Mariana Islands | MNP | Mar 31, 2020 | 0.937 | No | Mar 31, 2020 |
| 140 | Norway | NOR | Feb 27, 2020 | 0.835 | No | Feb 27, 2020 |
| 141 | Oman | OMN | Feb 25, 2020 | 0.928 | No | Feb 25, 2020 |
| 142 | Pakistan | PAK | Feb 27, 2020 | 0.728 | No | Feb 27, 2020 |
| 143 | Palestine | PSE | Mar 6, 2020 | 0.878 | No | Mar 6, 2020 |
| 144 | Panama | PAN | Mar 10, 2020 | 0.853 | No | Mar 10, 2020 |
| 145 | Paraguay | PRY | Mar 8, 2020 | 0.663 | No | Mar 8, 2020 |
| 146 | Peru | PER | Mar 7, 2020 | 0.701 | No | Mar 7, 2020 |
| 147 | Philippines | PHL | Jan 30, 2020 | 0.848 | No | Jan 30, 2020 |
| 148 | Poland | POL | Mar 4, 2020 | 0.590 | No | Mar 4, 2020 |
| 149 | Portugal | PRT | Mar 3, 2020 | 0.835 | No | Mar 3, 2020 |
| 150 | Puerto Rico | PRI | Mar 28, 2020 | 0.835 | No | Mar 28, 2020 |
| 151 | Qatar | QAT | Mar 1, 2020 | 0.878 | No | Mar 1, 2020 |
| 152 | Romania | ROU | Feb 27, 2020 | 0.744 | No | Feb 27, 2020 |
| 153 | Russia | RUS | Feb 1, 2020 | 0.046 | Yes | Mar 3, 2020 |
| 154 | Rwanda | RWA | Mar 15, 2020 | 0.866 | No | Mar 15, 2020 |
| 155 | Saint Kitts and Nevis | KNA | Mar 26, 2020 | 0.793 | No | Mar 26, 2020 |
| 156 | Saint Lucia | LCA | Mar 15, 2020 | 0.729 | No | Mar 15, 2020 |
| 157 | Saint Vincent and the Grenadines | VCT | Mar 13, 2020 | 0.421 | No | Mar 13, 2020 |
| 158 | San Marino | SMR | Feb 28, 2020 | 0.603 | No | Feb 28, 2020 |
| 159 | Sao Tome and Principe | STP | Apr 9, 2020 | 0.245 | No | Apr 9, 2020 |
| 160 | Saudi Arabia | SAU | Mar 3, 2020 | 0.654 | No | Mar 3, 2020 |
| 161 | Senegal | SEN | Mar 3, 2020 | 0.776 | No | Mar 3, 2020 |
| 162 | Serbia | SRB | Mar 7, 2020 | 0.499 | No | Mar 7, 2020 |
| 163 | Seychelles | SYC | Mar 15, 2020 | 0.966 | No | Mar 15, 2020 |
| 164 | Sierra Leone | SLE | Apr 1, 2020 | 0.808 | No | Apr 1, 2020 |
| 165 | Singapore | SGP | Jan 24, 2020 | 0.776 | No | Jan 24, 2020 |
| 166 | Sint Maarten (Dutch part) | SXM | Mar 3, 2020 | 0.216 | No | Mar 3, 2020 |
| 167 | Slovakia | SVK | Mar 7, 2020 | 0.845 | No | Mar 7, 2020 |
| 168 | Slovenia | SVN | Mar 5, 2020 | 0.851 | No | Mar 5, 2020 |
| 169 | Somalia | SOM | Mar 17, 2020 | 0.456 | No | Mar 17, 2020 |
| 170 | South Africa | ZAF | Mar 6, 2020 | 0.751 | No | Mar 6, 2020 |
| 171 | South Korea | KOR | Jan 20, 2020 | 0.620 | No | Jan 20, 2020 |
| 172 | South Sudan | SSD | Apr 6, 2020 | 0.822 | No | Apr 6, 2020 |
| 173 | Spain | ESP | Feb 1, 2020 | 0.390 | No | Feb 1, 2020 |
| 174 | Sri Lanka | LKA | Jan 28, 2020 | 0.007 | Yes | Mar 12, 2020 |
| 175 | Sudan | SDN | Mar 14, 2020 | 0.703 | No | Mar 14, 2020 |
| 176 | Suriname | SUR | Mar 15, 2020 | 0.579 | No | Mar 15, 2020 |
| 177 | Swaziland | SWZ | Mar 15, 2020 | 0.613 | No | Mar 15, 2020 |
| 178 | Sweden | SWE | Feb 1, 2020 | 0.024 | Yes | Feb 27, 2020 |
| 179 | Switzerland | CHE | Feb 26, 2020 | 0.701 | No | Feb 26, 2020 |
| 180 | Syria | SYR | Mar 23, 2020 | 0.768 | No | Mar 23, 2020 |
| 181 | Taiwan | TWN | Jan 21, 2020 | 0.677 | No | Jan 21, 2020 |
| 182 | Tanzania | TZA | Mar 17, 2020 | 0.846 | No | Mar 17, 2020 |
| 183 | Thailand | THA | Jan 13, 2020 | 0.803 | No | Jan 13, 2020 |
| 184 | Timor | TLS | Mar 22, 2020 | 0.190 | No | Mar 22, 2020 |
| 185 | Togo | TGO | Mar 7, 2020 | 0.144 | No | Mar 7, 2020 |
| 186 | Trinidad and Tobago | TTO | Mar 13, 2020 | 0.872 | No | Mar 13, 2020 |
| 187 | Tunisia | TUN | Mar 3, 2020 | 0.351 | No | Mar 3, 2020 |
| 188 | Turkey | TUR | Mar 12, 2020 | 0.848 | No | Mar 12, 2020 |
| 189 | Turks and Caicos Islands | TCA | Mar 25, 2020 | 0.927 | No | Mar 25, 2020 |
| 190 | Uganda | UGA | Mar 22, 2020 | 0.837 | No | Mar 22, 2020 |
| 191 | Ukraine | UKR | Mar 4, 2020 | 0.304 | No | Mar 4, 2020 |
| 192 | United Arab Emirates | ARE | Jan 27, 2020 | 0.863 | No | Jan 27, 2020 |
| 193 | United Kingdom | GBR | Jan 31, 2020 | 0.638 | No | Jan 31, 2020 |
| 194 | United States | USA | Jan 21, 2020 | 0.751 | No | Jan 21, 2020 |
| 195 | U.S. Virgin Islands | VIR | Mar 24, 2020 | 0.695 | No | Mar 24, 2020 |
| 196 | Uruguay | URY | Mar 15, 2020 | 0.835 | No | Mar 15, 2020 |
| 197 | Uzbekistan | UZB | Mar 16, 2020 | 0.861 | No | Mar 16, 2020 |
| 198 | Vatican | VAT | Mar 7, 2020 | 0.499 | No | Mar 7, 2020 |
| 199 | Venezuela | VEN | Mar 15, 2020 | 0.904 | No | Mar 15, 2020 |
| 200 | Vietnam | VNM | Jan 24, 2020 | 0.419 | No | Jan 24, 2020 |
| 201 | Zambia | ZMB | Mar 19, 2020 | 0.576 | No | Mar 19, 2020 |
| 202 | Zimbabwe | ZWE | Mar 21, 2020 | 0.933 | No | Mar 21, 2020 |

**Reference**

1. Solow AR, Smith W. How surprising is a new record? Am Stat. 2005;59: 153-155.
