# Supplementary material for: Dating first cases of COVID-19
Source: PLoS Pathog. 2021 Jun 24;17(6):e1009620. doi: 10.1371/journal.ppat.1009620 (PMC8224943; doi:10.1371/journal.ppat.1009620)
Supplement: S2 Table — (DOC) [file ppat.1009620.s002.doc]

**S2 Table.** Results of the COVID-19 origin dating per country, based on the Optimal Linear Estimation method.^1^ *N* - number of case dates used for the analysis, *t_k_* - duration of the interval between the first and the last case date used in the analysis (days), *θ* - estimated origin date, expressed as the number of days before the earliest reported case date, *S_U_* - upper bound of a 95% confidence interval of the estimated origin date.

| **No** | **Entity** | ***N*** | ***t_k_*** | ***θ*** | ***S_U_*** | **Covid origin date (*θ*)** | **Covid origin date (*S_U_*)** |
| --- | --- | --- | --- | --- | --- | --- | --- |
| 1 | Afghanistan | 10 | 29 | 14.9 | 59.6 | Feb 10, 2020 | Dec 27, 2019 |
| 2 | Albania | 10 | 10 | 1.5 | 5.8 | Mar 7, 2020 | Mar 3, 2020 |
| 3 | Algeria | 10 | 20 | 5.6 | 20.1 | Feb 20, 2020 | Feb 5, 2020 |
| 4 | Andorra | 10 | 23 | 13.0 | 54.4 | Feb 18, 2020 | Jan 8, 2020 |
| 5 | Angola | 10 | 19 | 6.4 | 23.2 | Mar 15, 2020 | Feb 27, 2020 |
| 6 | Antigua and Barbuda | 10 | 49 | 8.9 | 33.4 | Mar 6, 2020 | Feb 10, 2020 |
| 7 | Argentina | 10 | 13 | 2.5 | 9.2 | Mar 1, 2020 | Feb 23, 2020 |
| 8 | Armenia | 10 | 20 | 12.3 | 53.6 | Feb 17, 2020 | Jan 7, 2020 |
| 9 | Aruba | 10 | 23 | 8.7 | 32.4 | Mar 4, 2020 | Feb 9, 2020 |
| 10 | Australia | 10 | 21 | 2.0 | 8.4 | Jan 23, 2020 | Jan 16, 2020 |
| 11 | Austria | 10 | 11 | 2.5 | 8.9 | Feb 23, 2020 | Feb 17, 2020 |
| 12 | Azerbaijan | 10 | 21 | 4.3 | 15.8 | Feb 24, 2020 | Feb 13, 2020 |
| 13 | Bahamas | 10 | 19 | 5.8 | 21.0 | Mar 10, 2020 | Feb 23, 2020 |
| 14 | Bahrain | 10 | 13 | 1.5 | 6.0 | Feb 22, 2020 | Feb 18, 2020 |
| 15 | Bangladesh | 10 | 19 | 8.1 | 31.5 | Feb 29, 2020 | Feb 6, 2020 |
| 16 | Barbados | 10 | 14 | 2.4 | 8.9 | Mar 15, 2020 | Mar 9, 2020 |
| 17 | Belarus | 10 | 22 | 6.9 | 25.0 | Feb 21, 2020 | Feb 2, 2020 |
| 18 | Belgium | 10 | 10 | 1.5 | 5.8 | Feb 29, 2020 | Feb 25, 2020 |
| 19 | Belize | 10 | 20 | 5.7 | 21.0 | Mar 18, 2020 | Mar 3, 2020 |
| 20 | Benin | 10 | 25 | 5.8 | 21.1 | Mar 11, 2020 | Feb 24, 2020 |
| 21 | Bermuda | 10 | 19 | 4.5 | 16.5 | Mar 15, 2020 | Mar 3, 2020 |
| 22 | Bhutan | 7 | 61 | 17.7 | 77.9 | Feb 17, 2020 | Dec 19, 2019 |
| 23 | Bolivia | 10 | 13 | 2.1 | 8.1 | Mar 9, 2020 | Mar 3, 2020 |
| 24 | Bonaire Sint Eustatius and Saba | 5 | 34 | 18.5 | 118.9 | Mar 14, 2020 | Dec 5, 2019 |
| 25 | Bosnia and Herzegovina | 10 | 20 | 8.7 | 33.4 | Feb 26, 2020 | Feb 1, 2020 |
| 26 | Botswana | 9 | 35 | 2.6 | 12.3 | Mar 29, 2020 | Mar 19, 2020 |
| 27 | Brazil | 10 | 18 | 6.3 | 23.3 | Feb 19, 2020 | Feb 2, 2020 |
| 28 | British Virgin Islands | 6 | 40 | 11.8 | 62.2 | Mar 15, 2020 | Jan 24, 2020 |
| 29 | Brunei | 10 | 11 | 2.5 | 8.9 | Mar 7, 2020 | Mar 1, 2020 |
| 30 | Bulgaria | 10 | 12 | 2.5 | 9.2 | Mar 5, 2020 | Feb 27, 2020 |
| 31 | Burkina Faso | 10 | 16 | 5.3 | 19.2 | Mar 5, 2020 | Feb 20, 2020 |
| 32 | Burundi | 8 | 35 | 6.6 | 28.4 | Mar 25, 2020 | Mar 3, 2020 |
| 33 | Cambodia | 10 | 17 | 4.6 | 16.5 | Mar 3, 2020 | Feb 20, 2020 |
| 34 | Cameroon | 10 | 21 | 4.8 | 17.6 | Mar 2, 2020 | Feb 18, 2020 |
| 35 | Canada | 10 | 33 | 2.7 | 12.0 | Jan 23, 2020 | Jan 13, 2020 |
| 36 | Cape Verde | 10 | 27 | 3.3 | 13.2 | Mar 17, 2020 | Mar 7, 2020 |
| 37 | Cayman Islands | 10 | 17 | 4.0 | 15.2 | Mar 15, 2020 | Mar 4, 2020 |
| 38 | Central African Republic | 10 | 33 | 5.7 | 21.5 | Mar 10, 2020 | Feb 23, 2020 |
| 39 | Chad | 10 | 25 | 3.9 | 15.0 | Mar 16, 2020 | Mar 4, 2020 |
| 40 | Chile | 10 | 11 | 1.7 | 6.4 | Mar 2, 2020 | Feb 26, 2020 |
| 41 | China | 10 | 23 | 13.6 | 57.7 | Nov 17, 2019 | Oct 4, 2019 |
| 42 | Colombia | 10 | 14 | 4.5 | 16.2 | Mar 2, 2020 | Feb 19, 2020 |
| 43 | Congo | 10 | 31 | 5.7 | 21.2 | Mar 10, 2020 | Feb 23, 2020 |
| 44 | Costa Rica | 10 | 11 | 2.5 | 8.9 | Mar 4, 2020 | Feb 27, 2020 |
| 45 | Cote d'Ivoire | 10 | 15 | 4.2 | 15.0 | Mar 7, 2020 | Feb 25, 2020 |
| 46 | Croatia | 10 | 12 | 1.6 | 6.3 | Feb 24, 2020 | Feb 19, 2020 |
| 47 | Cuba | 10 | 14 | 4.1 | 15.1 | Mar 7, 2020 | Feb 25, 2020 |
| 48 | Curacao | 10 | 44 | 5.4 | 21.9 | Mar 7, 2020 | Feb 20, 2020 |
| 49 | Cyprus | 10 | 14 | 2.9 | 10.5 | Mar 7, 2020 | Feb 28, 2020 |
| 50 | Czech Republic | 10 | 11 | 2.0 | 7.5 | Feb 28, 2020 | Feb 23, 2020 |
| 51 | Democratic Republic of Congo | 10 | 20 | 4.9 | 17.6 | Mar 6, 2020 | Feb 22, 2020 |
| 52 | Denmark | 10 | 11 | 2.5 | 8.9 | Feb 24, 2020 | Feb 18, 2020 |
| 53 | Djibouti | 10 | 16 | 6.6 | 24.9 | Mar 12, 2020 | Feb 23, 2020 |
| 54 | Dominica | 8 | 44 | 2.5 | 11.6 | Mar 20, 2020 | Mar 11, 2020 |
| 55 | Dominican Republic | 10 | 24 | 8.8 | 32.4 | Feb 22, 2020 | Jan 29, 2020 |
| 56 | Ecuador | 10 | 15 | 1.7 | 6.9 | Feb 28, 2020 | Feb 23, 2020 |
| 57 | Egypt | 10 | 29 | 22.3 | 105.4 | Jan 23, 2020 | Nov 1, 2019 |
| 58 | El Salvador | 10 | 16 | 3.5 | 12.8 | Mar 15, 2020 | Mar 6, 2020 |
| 59 | Equatorial Guinea | 10 | 18 | 4.5 | 16.1 | Mar 10, 2020 | Feb 27, 2020 |
| 60 | Eritrea | 10 | 19 | 4.6 | 16.7 | Mar 17, 2020 | Mar 5, 2020 |
| 61 | Estonia | 10 | 18 | 6.8 | 25.1 | Feb 21, 2020 | Feb 2, 2020 |
| 62 | Ethiopia | 10 | 18 | 2.1 | 8.5 | Mar 11, 2020 | Mar 5, 2020 |
| 63 | Faeroe Islands | 10 | 10 | 1.5 | 5.8 | Mar 18, 2020 | Mar 14, 2020 |
| 64 | Falkland Islands | 6 | 32 | 3.7 | 22.0 | Mar 31, 2020 | Mar 13, 2020 |
| 65 | Fiji | 10 | 23 | 2.3 | 9.6 | Mar 17, 2020 | Mar 10, 2020 |
| 66 | Finland | 10 | 16 | 3.3 | 12.2 | Feb 23, 2020 | Feb 14, 2020 |
| 67 | France | 10 | 36 | 5.3 | 20.7 | Jan 19, 2020 | Jan 4, 2020 |
| 68 | French Polynesia | 10 | 13 | 2.3 | 8.6 | Mar 16, 2020 | Mar 10, 2020 |
| 69 | Gabon | 10 | 29 | 9.8 | 35.5 | Mar 3, 2020 | Feb 6, 2020 |
| 70 | Gambia | 9 | 49 | 8.5 | 33.9 | Mar 9, 2020 | Feb 13, 2020 |
| 71 | Georgia | 10 | 16 | 3.9 | 14.1 | Feb 23, 2020 | Feb 12, 2020 |
| 72 | Germany | 10 | 16 | 1.4 | 6.3 | Jan 26, 2020 | Jan 21, 2020 |
| 73 | Ghana | 10 | 14 | 5.1 | 18.7 | Mar 7, 2020 | Feb 23, 2020 |
| 74 | Gibraltar | 10 | 16 | 4.1 | 14.9 | Mar 15, 2020 | Mar 5, 2020 |
| 75 | Greece | 10 | 13 | 1.9 | 7.5 | Feb 25, 2020 | Feb 19, 2020 |
| 76 | Greenland | 7 | 47 | 3.7 | 17.7 | Mar 16, 2020 | Mar 2, 2020 |
| 77 | Grenada | 10 | 42 | 5.1 | 20.5 | Mar 17, 2020 | Mar 2, 2020 |
| 78 | Guam | 10 | 10 | 1.5 | 5.8 | Mar 17, 2020 | Mar 13, 2020 |
| 79 | Guatemala | 10 | 12 | 3.0 | 11.0 | Mar 11, 2020 | Mar 4, 2020 |
| 80 | Guernsey | 10 | 13 | 3.1 | 11.1 | Mar 16, 2020 | Mar 8, 2020 |
| 81 | Guinea | 10 | 24 | 9.9 | 37.1 | Mar 4, 2020 | Feb 5, 2020 |
| 82 | Guinea-Bissau | 10 | 20 | 5.2 | 18.7 | Mar 21, 2020 | Mar 8, 2020 |
| 83 | Guyana | 10 | 26 | 7.5 | 28.2 | Mar 5, 2020 | Feb 13, 2020 |
| 84 | Haiti | 10 | 20 | 4.7 | 17.0 | Mar 15, 2020 | Mar 2, 2020 |
| 85 | Honduras | 10 | 13 | 2.9 | 10.5 | Mar 9, 2020 | Mar 1, 2020 |
| 86 | Hungary | 10 | 11 | 2.5 | 8.9 | Mar 2, 2020 | Feb 25, 2020 |
| 87 | Iceland | 10 | 11 | 2.5 | 8.9 | Feb 26, 2020 | Feb 20, 2020 |
| 88 | India | 10 | 41 | 9.6 | 37.8 | Jan 20, 2020 | Dec 23, 2019 |
| 89 | Indonesia | 10 | 17 | 6.6 | 24.7 | Feb 24, 2020 | Feb 6, 2020 |
| 90 | Iran | 10 | 10 | 1.5 | 5.8 | Feb 18, 2020 | Feb 14, 2020 |
| 91 | Iraq | 10 | 11 | 2.0 | 7.5 | Feb 22, 2020 | Feb 17, 2020 |
| 92 | Ireland | 10 | 13 | 3.2 | 11.7 | Feb 26, 2020 | Feb 18, 2020 |
| 93 | Isle of Man | 10 | 13 | 2.0 | 7.8 | Mar 18, 2020 | Mar 13, 2020 |
| 94 | Israel | 10 | 17 | 4.4 | 16.1 | Feb 17, 2020 | Feb 5, 2020 |
| 95 | Italy | 10 | 10 | 1.5 | 5.8 | Feb 20, 2020 | Feb 16, 2020 |
| 96 | Jamaica | 10 | 15 | 1.6 | 6.7 | Mar 10, 2020 | Mar 5, 2020 |
| 97 | Japan | 10 | 20 | 11.2 | 46.6 | Jan 3, 2020 | Nov 29, 2019 |
| 98 | Jersey | 10 | 16 | 1.9 | 7.6 | Mar 18, 2020 | Mar 12, 2020 |
| 99 | Jordan | 10 | 22 | 15.0 | 68.1 | Feb 17, 2020 | Dec 25, 2019 |
| 100 | Kazakhstan | 10 | 10 | 1.5 | 5.8 | Mar 13, 2020 | Mar 9, 2020 |
| 101 | Kenya | 10 | 17 | 3.3 | 12.0 | Mar 10, 2020 | Mar 1, 2020 |
| 102 | Kosovo | 10 | 13 | 3.3 | 11.7 | Mar 12, 2020 | Mar 4, 2020 |
| 103 | Kuwait | 10 | 14 | 1.2 | 5.2 | Feb 22, 2020 | Feb 18, 2020 |
| 104 | Kyrgyzstan | 10 | 15 | 2.0 | 7.9 | Mar 16, 2020 | Mar 11, 2020 |
| 105 | Laos | 10 | 20 | 4.7 | 17.1 | Mar 20, 2020 | Mar 7, 2020 |
| 106 | Latvia | 10 | 14 | 5.4 | 20.2 | Feb 26, 2020 | Feb 11, 2020 |
| 107 | Lebanon | 10 | 18 | 5.3 | 19.3 | Feb 16, 2020 | Feb 2, 2020 |
| 108 | Liberia | 10 | 26 | 4.8 | 18.6 | Mar 12, 2020 | Feb 27, 2020 |
| 109 | Libya | 10 | 21 | 6.1 | 22.1 | Mar 18, 2020 | Mar 2, 2020 |
| 110 | Liechtenstein | 10 | 22 | 8.5 | 31.6 | Feb 25, 2020 | Feb 2, 2020 |
| 111 | Lithuania | 10 | 23 | 15.5 | 69.5 | Feb 12, 2020 | Dec 20, 2019 |
| 112 | Luxembourg | 10 | 19 | 7.8 | 29.2 | Feb 22, 2020 | Jan 31, 2020 |
| 113 | Macedonia | 10 | 24 | 12.7 | 51.3 | Feb 14, 2020 | Jan 6, 2020 |
| 114 | Madagascar | 10 | 13 | 3.8 | 13.8 | Mar 17, 2020 | Mar 7, 2020 |
| 115 | Malawi | 10 | 20 | 2.4 | 9.5 | Mar 31, 2020 | Mar 24, 2020 |
| 116 | Malaysia | 10 | 17 | 3.3 | 12.3 | Jan 21, 2020 | Jan 12, 2020 |
| 117 | Maldives | 10 | 25 | 1.9 | 8.6 | Mar 6, 2020 | Feb 28, 2020 |
| 118 | Mali | 10 | 13 | 2.3 | 8.7 | Mar 23, 2020 | Mar 17, 2020 |
| 119 | Malta | 10 | 12 | 3.0 | 11.0 | Mar 4, 2020 | Feb 26, 2020 |
| 120 | Mauritania | 7 | 52 | 6.0 | 32.6 | Mar 8, 2020 | Feb 11, 2020 |
| 121 | Mauritius | 10 | 10 | 1.5 | 5.8 | Mar 18, 2020 | Mar 14, 2020 |
| 122 | Mexico | 10 | 18 | 3.1 | 12.0 | Feb 25, 2020 | Feb 17, 2020 |
| 123 | Moldova | 10 | 12 | 3.4 | 12.3 | Mar 4, 2020 | Feb 24, 2020 |
| 124 | Monaco | 10 | 27 | 16.8 | 73.4 | Feb 12, 2020 | Dec 17, 2019 |
| 125 | Mongolia | 10 | 31 | 6.9 | 25.5 | Mar 3, 2020 | Feb 13, 2020 |
| 126 | Montenegro | 10 | 11 | 1.7 | 6.4 | Mar 16, 2020 | Mar 11, 2020 |
| 127 | Montserrat | 5 | 46 | 12.9 | 80.5 | Mar 8, 2020 | Dec 31, 2019 |
| 128 | Morocco | 10 | 17 | 6.4 | 24.2 | Feb 25, 2020 | Feb 7, 2020 |
| 129 | Mozambique | 10 | 24 | 4.4 | 16.2 | Mar 18, 2020 | Mar 6, 2020 |
| 130 | Myanmar | 10 | 15 | 2.1 | 8.3 | Mar 21, 2020 | Mar 15, 2020 |
| 131 | Namibia | 8 | 52 | 9.3 | 37.7 | Mar 5, 2020 | Feb 6, 2020 |
| 132 | Nepal | 10 | 27 | 4.2 | 16.2 | Mar 19, 2020 | Mar 7, 2020 |
| 133 | Netherlands | 10 | 10 | 1.5 | 5.8 | Feb 26, 2020 | Feb 22, 2020 |
| 134 | New Caledonia | 9 | 46 | 1.0 | 5.1 | Mar 20, 2020 | Mar 15, 2020 |
| 135 | New Zealand | 10 | 22 | 5.3 | 19.2 | Feb 22, 2020 | Feb 8, 2020 |
| 136 | Nicaragua | 10 | 39 | 6.4 | 24.3 | Mar 12, 2020 | Feb 23, 2020 |
| 137 | Niger | 10 | 18 | 4.3 | 15.7 | Mar 16, 2020 | Mar 5, 2020 |
| 138 | Nigeria | 10 | 28 | 18.3 | 81.0 | Feb 9, 2020 | Dec 8, 2019 |
| 139 | Northern Mariana Islands | 6 | 36 | 2.4 | 15.5 | Mar 28, 2020 | Mar 15, 2020 |
| 140 | Norway | 10 | 10 | 1.5 | 5.8 | Feb 25, 2020 | Feb 21, 2020 |
| 141 | Oman | 10 | 22 | 2.5 | 10.3 | Feb 22, 2020 | Feb 14, 2020 |
| 142 | Pakistan | 10 | 20 | 5.6 | 20.4 | Feb 21, 2020 | Feb 6, 2020 |
| 143 | Palestine | 10 | 13 | 1.7 | 6.6 | Mar 4, 2020 | Feb 28, 2020 |
| 144 | Panama | 10 | 11 | 1.5 | 5.9 | Mar 8, 2020 | Mar 4, 2020 |
| 145 | Paraguay | 10 | 15 | 4.1 | 14.7 | Mar 3, 2020 | Feb 22, 2020 |
| 146 | Peru | 10 | 11 | 2.5 | 8.9 | Mar 4, 2020 | Feb 27, 2020 |
| 147 | Philippines | 10 | 45 | 11.2 | 44.5 | Jan 18, 2020 | Dec 16, 2019 |
| 148 | Poland | 10 | 12 | 3.4 | 12.3 | Feb 29, 2020 | Feb 20, 2020 |
| 149 | Portugal | 10 | 10 | 1.5 | 5.8 | Mar 1, 2020 | Feb 26, 2020 |
| 150 | Puerto Rico | 10 | 10 | 1.5 | 5.8 | Mar 26, 2020 | Mar 22, 2020 |
| 151 | Qatar | 10 | 14 | 2.4 | 8.9 | Feb 27, 2020 | Feb 21, 2020 |
| 152 | Romania | 10 | 15 | 4.5 | 16.4 | Feb 22, 2020 | Feb 10, 2020 |
| 153 | Russia | 10 | 16 | 3.5 | 13.1 | Feb 28, 2020 | Feb 18, 2020 |
| 154 | Rwanda | 10 | 13 | 2.3 | 8.4 | Mar 12, 2020 | Mar 6, 2020 |
| 155 | Saint Kitts and Nevis | 9 | 41 | 4.2 | 16.9 | Mar 21, 2020 | Mar 9, 2020 |
| 156 | Saint Lucia | 9 | 52 | 8.1 | 33.5 | Mar 6, 2020 | Feb 10, 2020 |
| 157 | Saint Vincent and the Grenadines | 10 | 53 | 21.0 | 78.9 | Feb 20, 2020 | Dec 25, 2019 |
| 158 | San Marino | 10 | 13 | 4.0 | 14.7 | Feb 23, 2020 | Feb 13, 2020 |
| 159 | Sao Tome and Principe | 8 | 27 | 18.4 | 83.4 | Mar 21, 2020 | Jan 16, 2020 |
| 160 | Saudi Arabia | 10 | 14 | 3.7 | 13.3 | Feb 28, 2020 | Feb 18, 2020 |
| 161 | Senegal | 10 | 17 | 4.2 | 15.5 | Feb 27, 2020 | Feb 16, 2020 |
| 162 | Serbia | 10 | 13 | 4.4 | 16.0 | Mar 2, 2020 | Feb 19, 2020 |
| 163 | Seychelles | 8 | 52 | 1.1 | 8.1 | Mar 13, 2020 | Mar 6, 2020 |
| 164 | Sierra Leone | 10 | 17 | 3.2 | 11.7 | Mar 28, 2020 | Mar 20, 2020 |
| 165 | Singapore | 10 | 14 | 2.6 | 9.7 | Jan 21, 2020 | Jan 14, 2020 |
| 166 | Sint Maarten (Dutch part) | 10 | 43 | 24.0 | 101.0 | Feb 7, 2020 | Nov 23, 2019 |
| 167 | Slovakia | 10 | 11 | 1.8 | 6.9 | Mar 5, 2020 | Feb 29, 2020 |
| 168 | Slovenia | 10 | 11 | 1.6 | 6.1 | Mar 3, 2020 | Feb 27, 2020 |
| 169 | Somalia | 10 | 30 | 11.9 | 44.6 | Mar 5, 2020 | Feb 1, 2020 |
| 170 | South Africa | 10 | 13 | 2.5 | 9.3 | Mar 3, 2020 | Feb 25, 2020 |
| 171 | South Korea | 10 | 18 | 5.5 | 19.9 | Jan 14, 2020 | Dec 31, 2019 |
| 172 | South Sudan | 10 | 29 | 5.3 | 19.9 | Mar 31, 2020 | Mar 17, 2020 |
| 173 | Spain | 10 | 32 | 19.6 | 89.4 | Jan 12, 2020 | Nov 3, 2019 |
| 174 | Sri Lanka | 10 | 11 | 1.5 | 5.7 | Mar 10, 2020 | Mar 6, 2020 |
| 175 | Sudan | 10 | 29 | 8.6 | 31.3 | Mar 5, 2020 | Feb 11, 2020 |
| 176 | Suriname | 5 | 52 | 9.1 | 56.5 | Mar 5, 2020 | Jan 18, 2020 |
| 177 | Swaziland | 10 | 35 | 11.2 | 40.8 | Mar 3, 2020 | Feb 3, 2020 |
| 178 | Sweden | 10 | 10 | 1.5 | 5.8 | Feb 25, 2020 | Feb 21, 2020 |
| 179 | Switzerland | 10 | 11 | 2.5 | 8.9 | Feb 23, 2020 | Feb 17, 2020 |
| 180 | Syria | 10 | 27 | 5.3 | 19.6 | Mar 17, 2020 | Mar 3, 2020 |
| 181 | Taiwan | 10 | 19 | 4.3 | 15.8 | Jan 16, 2020 | Jan 5, 2020 |
| 182 | Tanzania | 10 | 21 | 3.6 | 13.6 | Mar 13, 2020 | Mar 3, 2020 |
| 183 | Thailand | 10 | 31 | 5.9 | 21.8 | Jan 7, 2020 | Dec 22, 2019 |
| 184 | Timor | 9 | 45 | 24.3 | 109.5 | Feb 26, 2020 | Dec 3, 2019 |
| 185 | Togo | 10 | 25 | 17.0 | 76.7 | Feb 18, 2020 | Dec 21, 2019 |
| 186 | Trinidad and Tobago | 10 | 13 | 2.0 | 7.6 | Mar 11, 2020 | Mar 5, 2020 |
| 187 | Tunisia | 10 | 17 | 7.3 | 28.3 | Feb 24, 2020 | Feb 3, 2020 |
| 188 | Turkey | 10 | 12 | 2.5 | 9.2 | Mar 9, 2020 | Mar 2, 2020 |
| 189 | Turks and Caicos Islands | 8 | 42 | 3.5 | 17.9 | Mar 21, 2020 | Mar 7, 2020 |
| 190 | Uganda | 10 | 19 | 3.1 | 11.7 | Mar 18, 2020 | Mar 10, 2020 |
| 191 | Ukraine | 10 | 22 | 12.0 | 49.4 | Feb 20, 2020 | Jan 14, 2020 |
| 192 | United Arab Emirates | 10 | 35 | 5.7 | 21.6 | Jan 21, 2020 | Jan 5, 2020 |
| 193 | United Kingdom | 10 | 32 | 8.8 | 31.7 | Jan 22, 2020 | Dec 30, 2019 |
| 194 | United States | 10 | 24 | 4.6 | 17.2 | Jan 16, 2020 | Jan 3, 2020 |
| 195 | United States Virgin Islands | 10 | 20 | 4.3 | 15.5 | Mar 19, 2020 | Mar 8, 2020 |
| 196 | Uruguay | 10 | 10 | 1.5 | 5.8 | Mar 13, 2020 | Mar 9, 2020 |
| 197 | Uzbekistan | 10 | 12 | 1.7 | 6.7 | Mar 14, 2020 | Mar 9, 2020 |
| 198 | Vatican | 8 | 60 | 22.7 | 92.2 | Feb 13, 2020 | Dec 5, 2019 |
| 199 | Venezuela | 10 | 17 | 2.2 | 8.8 | Mar 12, 2020 | Mar 6, 2020 |
| 200 | Vietnam | 10 | 19 | 7.5 | 28.3 | Jan 16, 2020 | Dec 26, 2019 |
| 201 | Zambia | 10 | 16 | 5.4 | 19.7 | Mar 13, 2020 | Feb 28, 2020 |
| 202 | Zimbabwe | 10 | 23 | 2.7 | 11.0 | Mar 18, 2020 | Mar 10, 2020 |

**Reference**

1. Roberts DL, Solow AR. When did the dodo become extinct? Nature 2003;426: 245.
