# Supplementary material for: Dating first cases of COVID-19
Source: PLoS Pathog. 2021 Jun 24;17(6):e1009620. doi: 10.1371/journal.ppat.1009620 (PMC8224943; doi:10.1371/journal.ppat.1009620)
Supplement: S3 Table — N—number of case dates used for the analysis, tk—duration of the interval between the first and the last case date used in the analysis (days), θ - estimated origin date, expressed as the number of days before the earliest reported case date, SU—upper bound of a 95% confidence interval of the estimated origin date. (DOC) [file ppat.1009620.s003.doc]

**S3 Table.** Results of the COVID-19 origin dating without the correction of the first case date, for the nine countries that had their first case dates adjusted (see Methods for information). *N* - number of case dates used for the analysis, *t_k_* - duration of the interval between the first and the last case date used in the analysis (days), *θ* - estimated origin date, expressed as the number of days before the earliest reported case date, *S_U_* - upper bound of a 95% confidence interval of the estimated origin date.

| **No** | **Entity** | **First case date (uncorrected)** | ***N*** | ***t_k_*** | ***θ*** | ***S_U_*** | **Covid origin date (*θ*)** | **Covid origin date (*S_U_*)** |
| --- | --- | --- | --- | --- | --- | --- | --- | --- |
| 1 | Belgium | Feb 4, 2020 | 10 | 36 | 39.1 | 215.5 | Dec 26, 2019 | Jul 3, 2019 |
| 2 | Cambodia | Jan 28, 2020 | 10 | 56 | 58.3 | 313.6 | Nov 30, 2019 | Mar 20, 2019 |
| 3 | Finland | Jan 30, 2020 | 10 | 43 | 35.3 | 172.3 | Dec 25, 2019 | Aug 10, 2019 |
| 4 | Italy | Jan 31, 2020 | 10 | 31 | 29.4 | 153.5 | Jan 1, 2020 | Aug 30, 2019 |
| 5 | Nepal | Jan 25, 2020 | 10 | 85 | 77.6 | 393.5 | Nov 8, 2019 | Dec 27, 2018 |
| 6 | Russia | Feb 1, 2020 | 10 | 46 | 44.9 | 234.1 | Dec 18, 2019 | Jun 11, 2019 |
| 7 | Sri Lanka | Jan 28, 2020 | 10 | 54 | 72.6 | 430.8 | Nov 16, 2019 | Nov 23, 2018 |
| 8 | Sweden | Feb 1, 2020 | 10 | 35 | 37.1 | 202.3 | Dec 25, 2019 | Jul 13, 2019 |
| 9 | Yemen | Apr 10, 2020 | 5 | 26 | 56.5 | 437.1 | Feb 13, 2020 | Jan 28, 2019 |
